# The mechanisms of chromogranin B-regulated Cl^−^ homeostasis

**DOI:** 10.1042/BST20220435

**Published:** 2022-12-13

**Authors:** Qiu-Xing Jiang, Gaya Yadav

**Affiliations:** 1 Department of Physiology and Biophysics, University at Buffalo, Buffalo, NY 14210, U.S.A.; 2 Department of Medicinal Chemistry, University of Florida, Gainesville, FL 32610, U.S.A.; 3 Laboratory of Molecular Physiology and Biophysics, Hauptman-Woodward Medical Research Institute, Buffalo, NY 14230, U.S.A.; 4 Department of Biochemistry and Biophysics, Texas A&M University, College Station, TX, U.S.A.

**Keywords:** anion shunting pathway, chloride and cation storage, chloride loaders and extruders, excitable membranes, inhibitory and excitatory signals, regulated secretion

## Abstract

Chloride is the most abundant inorganic anions in almost all cells and in human circulation systems. Its homeostasis is therefore important for systems physiology and normal cellular activities. This topic has been extensively studied with chloride loaders and extruders expressed in both cell surfaces and intracellular membranes. With the newly discovered, large-conductance, highly selective Cl^−^ channel formed by membrane-bound chromogranin B (CHGB), which differs from all other known anion channels of conventional transmembrane topology, and is distributed in plasma membranes, endomembrane systems, endosomal, and endolysosomal compartments in cells expressing it, we will discuss the potential physiological importance of the CHGB channels to Cl^−^ homeostasis, cellular excitability and volume control, and cation uptake or release at the cellular and subcellular levels. These considerations and CHGB's association with human diseases make the CHGB channel a possible druggable target for future molecular therapeutics.

## Introduction

Chloride is probably the most abundant inorganic anion in all living organisms [5,6]. Even though Cl^−^ flux has often been viewed as an accompanying event auxiliary to cation-dominating currents [7–9], such as Ca^2+^ inflow at the presynaptic terminals prior to release of synaptic vesicles and H^+^-flow across secretory granular or endosomal membranes [3,10–14], the resting concentration of intracellular [Cl^−^] can be one of the key determinants for cellular excitability in certain cells, such as various types of neurons, muscle cells and neuroendocrine cells [15]. Influx of Cl^−^ through GABAergic or glycinergic receptors in central nervous system (CNS) is a classic inhibitory signal because intracellular Cl^−^ concentration, [Cl^−^]_i_, in neurons approached by earlier studies is low at the resting state [16], making the Nernst potential of Cl^−^ (*E_Cl_*) lower than the resting transmembrane electrostatic potential (Vm) and driving Cl^−^ influx upon channel opening, and the negative charges via Cl^−^ influx will hyperpolarize *Vm* and decrease excitability. On the other hand, in cells with elevated [Cl^−^]_i_, such as ∼34 mM in pancreatic beta-cells and ∼85 mM in (murine) vomeronasal neurons [17,18], the resting *Vm* is lower (less negative) than *E_Cl_*, which will drive Cl^−^ efflux through Cl^−^ channels and therefore produce excitatory signals. In peripheral dorsal root ganglion (DRG) neurons, [Cl^−^]_i_ may vary so that their reversal potentials of Cl^−^ may change between −20 and −70 mV and either excitatory or inhibitory signals are possible [19]. The difference between inhibitory and excitatory signaling is fundamentally important to physiological controls at the cellular and systems levels, which means that proper regulation of resting [Cl^−^]_i_ in different cell types, especially excitable ones, is critical for Cl^−^ homeostasis and for cellular physiology. Among different types of human cells, resting [Cl^−^]_i_ can vary in a broad range of ∼5 mM to 80 mM. Cl^−^ homeostasis at the cellular and systems levels is critically important, especially from the angle of anion homeostasis and signaling through other cations like Ca^2+^. At the systems level, the Cl^−^ concentration is a balance chiefly between Cl^−^ intake through the digestive system and Cl^−^ excretion via the urinary and digestive systems, where different hormones and cells play important roles in intricate feedback loops. In the next, we will focus on the Cl^−^ homeostasis at the cellular level and in cells that express chromogranin B (CHGB) protein. The [Cl^−^]_i_ ought to be balanced with other anions such as HCO_3_^−^, NO_3_^−^, taurine, etc., via the actions of anion exchangers and various Cl^−^ channels permeable to them. However, in this short review, we will not discuss these other anions because of their nondetectable permeation through the CHGB channels, instead of referring readers to other studies (such as refs. [20–27]).

**Figure 1.**
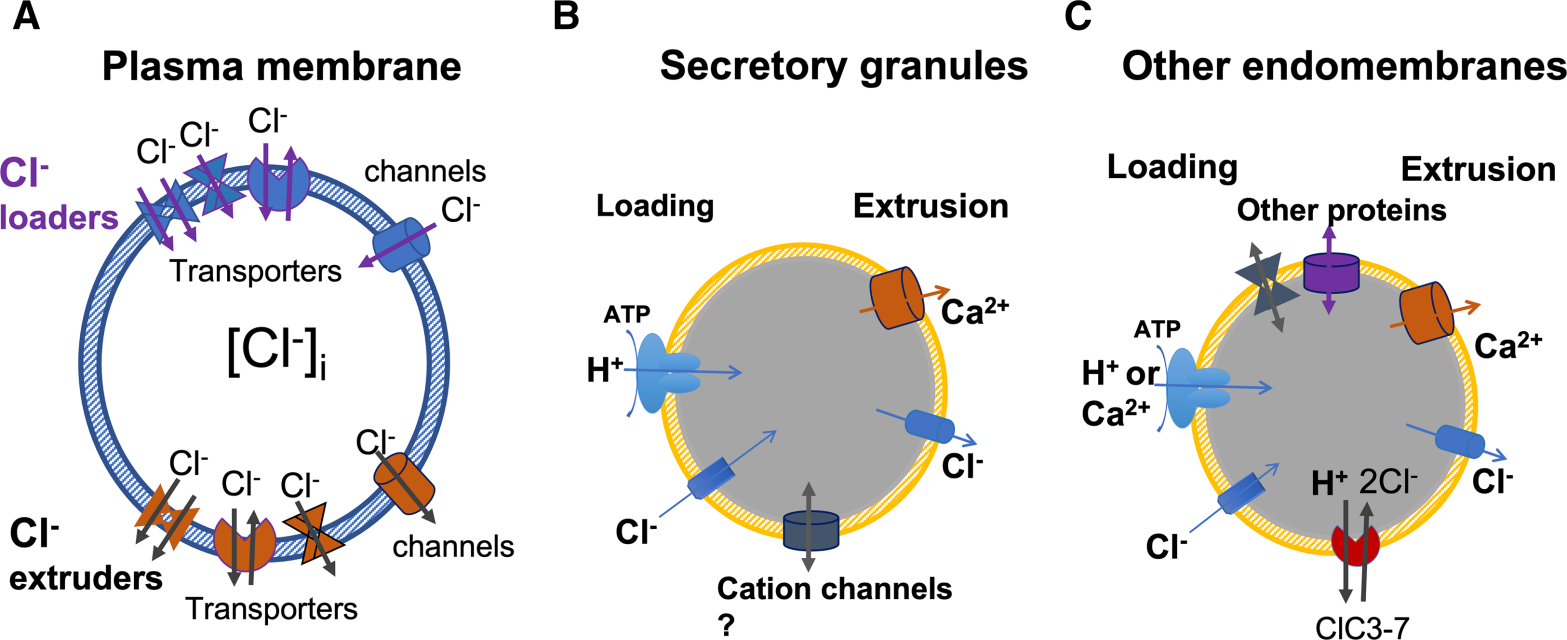
Chloride loading and extrusion on cell surface or in certain intracellular membranes. (**A**) In plasma membranes, transporters and chloride channels act as Cl^−^ loaders or extruders depending electrochemical conditions. The collective actions of these proteins may keep the intracellular Cl^−^ concentration above or below the level determined by the resting transmembrane potential (Vm). The Nernst potential of Cl^−^ may be higher or lower than Vm. (**B**) In secretory granules, proton pumping and Cl^−^ shunt load the granules to enable granule acidification. Ca^2+^ release from the granules is accompanied by Cl^−^ release. The participation of other cation channels is still debated because of uncertainty in their presence in granular membranes. (**C**) In other intracellular compartments, including ER, Golgi, endosomes and lysosomes, Ca^2+^ or H^+^ usually is loaded by pumps consuming energy. Cl^−^ or other ions are needed to move across the membranes to nullify charge accumulation and fill the stores. Quick dumping of the stores needs cation or anion fluxes to balance charges.

Cl^−^ homeostasis is mediated by Cl^−^ transporters and channels in general (Figure 1) [18]. These proteins inside the plasma membranes can be classified as Cl^−^ loaders and Cl^−^ extruders, depending on whether they move Cl^−^ into the cells (influx or inflow) or out (efflux or outflow). A key difference is that the Cl^−^ channels are passive action-takers because they let the anions flow down their electrochemical gradients (Figure 1A). Depending on ionic conditions and Vm across the plasma membranes, a transporter or channel may be a loader or an extruder under different situations. The Cl^−^ transporters include Na^+^-K^+^-2Cl^−^ cotransporter 1 and 2 (NKCC1/2) [28], K^+^-Cl^−^ cotransporter (KCC) [29], Na^+^-Cl^−^ cotransporter (NCC) [18,30,31], CLC family transporters (2Cl^−^/H^+^) [32–34], Cl^−^/bicarbonate exchangers (CBE, both SLC26 and anion exchangers 1–3 [AE1–3]) [15,19,22], etc. The chloride channels on the cell surface consist of a diversified group, including different families of proteins, such as Cystic Fibrosis Transmembrane Regulator (CFTR) protein [18,35,36], CLC-family anion channels (CLC-1, -2, -Ka, or -Kb) [37–39], calcium-activated chloride channels (CACCs; TMEM16A/B/F) [40–42], acid-activated Cl channels (PACC1 or TMEM206) [43,44], bestrophin family proteins [45–48], glutamate-gated chloride channels (GluCl) [49,50], Gly/GABA receptors [16,51–54], F^−^-selective channels (Fluc channels) [55–58], volume-regulated anion channels (VRAC; LRRC8A subfamily proteins) [59–62], voltage-dependent anion channels (VDAC) [63,64], CHGB subfamily [2,4], etc. There are other electrophysiologically identified Cl^−^ channels whose genetic identity remains unclear [41].

Among all Cl^−^-conducting channels, the CHGB channel is so far the only one that has a large conductance (∼140 pS in the normal salt conditions), shows very strong anion selectivity, F^−^ ∼ Cl^−^ >> (Br^−^, I^−^, NO_3_^−^, SCN^−^, etc.), instead of (Br^−^, I^−^) > Cl^−^, and exhibits high sensitivity to DIDS (*K*_d_ ∼ 0.5 µM vs. ∼100 µM for CLC-family proteins). Its unconventional activities were first monitored through recordings of Cl^−^ release from KCl^−^loaded CHGB-vesicles by an Ag/AgCl electrode, with flux assays that measure activities of CHGB in billions of vesicles each time by both steady-state and stopped-flow fluorimetry, and in lipid bilayers containing a couple to dozens of channels per membrane [4]. In all these assays, the high purity of CHGB protein (∼99.8%) made it unequivocal to assign the channel functions to the CHGB protein because the probability for any residual contaminant protein to do so was <1.0 × 10^−7^. The uncanonical transmembrane topology of CHGB remains undefined, is reminiscent of a plethora of dimorphic proteins [65], whose soluble forms undergo conformational changes to reconstitute oligomeric channels in membrane, and will need high-resolution structures for full demonstration. The membrane-integrated state of the CHGB is also consistent with the ‘tightly membrane-bound form' of full-length CHGB that was delivered to the surface of PC-12 cells after stimulated granule release, endocytosed 2–4 h later and resistant to harsh conditions for membrane-dissociation except detergents [66]. Furthermore, the CHGB Cl^−^ channel activity in secretory granules provides a simple explanation of earlier observations of impaired proinsulin-insulin conversion in pancreatic beta-cells and decreased monoamine content in adrenal chromaffin cells from CHGB^−/−^ mice [67–69]. In retrospect, the strict Cl^−^ selectivity of CHGB channels in the secretory granules over HCO_3_^−^, NO_3_^−^, sulfate, phosphate, taurine, etc., is physiologically desired in order to prevent these anions and other organic anions including formate, ADP, pyruvate, oxalate, and lactate from being concentrated into the granules and wasted via granule release [2,4]. The expression levels and distribution patterns of these transporters and channels may vary among different cell types or with time in the same cell type, making it feasible for the cells to change their intracellular Cl^−^ levels.

Negative resting transmembrane potential usually favors a low intracellular [Cl^−^ ] of 5–15 mM in many cells. In these cells, the Nernst potential of Cl^−^ is close to Vm and there is not much Cl^−^ flux through channels or moved by transporters, which is energetically economic [15,18]. The Cl^−^ transporters may work against the resting Vm, and thus increase or decrease the [Cl^−^]_i_ so that the Nernst potential for Cl^−^ is away from the Vm. If so, the difference between the Cl^−^ Nernst potential and Vm will drive anion flow through the channels. Under such conditions, the flux through the channels should be minimal so that a stable [Cl^−^]_i_ level can be established without a significant waste of energy. This may be a reason why nearly all Cl^−^ channels on the cell surface show small (or tiny) single channel conductances (*g*) when Vm lies in [−60,−10] mV and at a relatively low abundancy per cell in order to prevent Cl^−^ ions from being equilibrated quickly and keep the Cl Nernst potential stably away from the resting Vm. For example, in a typical cell of ∼20–30 microns in diameter, a cytosolic [Cl^−^] of ∼34 mM (as in a beta-cell) takes ∼9.0 × 10^10^ free ions, which is a significant amount, and will take a long period of time for a small conductance Cl^−^ channel to pass (e.g. ∼4000 seconds for a 5.0 pS channel with a driving force of 50 mV). Another layer of control is that the number of the chloride channels on the cell surface (*N*), their open probability (*P_o_*), and their modulation by various factors to change the total conductance (*G_Cl_* = *N* × *g* × *P_o_*) may all be controlled and utilized so that a minimal Cl^−^ conductance (G_Cl_) is present and helps maintain a high Cl^−^ concentration inside without a significant energy cost. Consistently, it is interesting to note that human ClC-1 has a single channel conductance less than 1.5 pS [70], drosophila bestrophin 1 (dBEST1) ∼2 pS [71], human bestrophin <1 pS [72], and ClC-2 ∼5 pS [73]; and their open probability vary when Vm is in the range of [−60, 0 mV], probably for the very purpose of enabling cells to maintain their [Cl^−^]_i_ away from what is dictated by their resting Vm. Under the same logic, when a cell adjusts its volume, it will move large amounts of anions across the plasma membranes in a short period of time, and thus need to express and deliver or turn on a large conductance by summing Cl^−^ channels or transporters with high turnover rates in order to quickly satisfy the needs.

Inside the cells, Cl^−^ concentrations may be higher in various organelles, such as ER, Golgi apparatus, secretory granules, etc. (Figure 1B). The high intracellular stock of Ca^2+^ (total ∼20 mM) needs anions, both free and fixed ones, to balance charges, which probably include a significant fraction of Cl^−^. For organelles with strong acidic pH driven by proton-ATPase, a Cl^−^ loader, either a transporter or a channel, and/or a counterflow of cations is important for quick charge neutralization and fast acidification of luminal contents inside [3,13]. For organelles acting as calcium stores, pumping of Ca^2+^ into the luminal side or release of Ca^2+^ from the store also will happen quickly when Cl^−^ anions or cations are able to move from one side of the membrane to the other (ref. [74]) so that no charge accumulation in the organelles would counteract against the Ca^2+^ relocation (Figure 1B,C) [75].

From a physiological standing point, the anion homeostasis is well understood for Cl^−^ flows across plasma membranes when the influx and efflux reach a steady-state equilibrium. Multiple reviews have been written for different systems [15,18,19]. However, the picture would be somewhat different if there are large-conductance Cl^−^-selective channels on the cell surface or in the intracellular membranes (Figure 1B). With the large-conductance (∼150 pS in physiological ionic conditions), highly selective Cl^−^ channel reconstituted by an obligate secretory granule protein, CHGB, we can and need to view anion homeostasis from a different perspective by bringing the CHGB channel into the play, which will be the focus of the discussions in the next sections. Our discussions are expectedly applicable to all CHGB-expressing cells, including exocrine, endocrine, neural and stem cells. and may be relevant to primary neuroendocrine tumors and neuroendocrine transforms of various types of cancer cells as well [76,77].

## Anion shunting in regulated secretion and the CHGB anion channel

**Figure 2.**
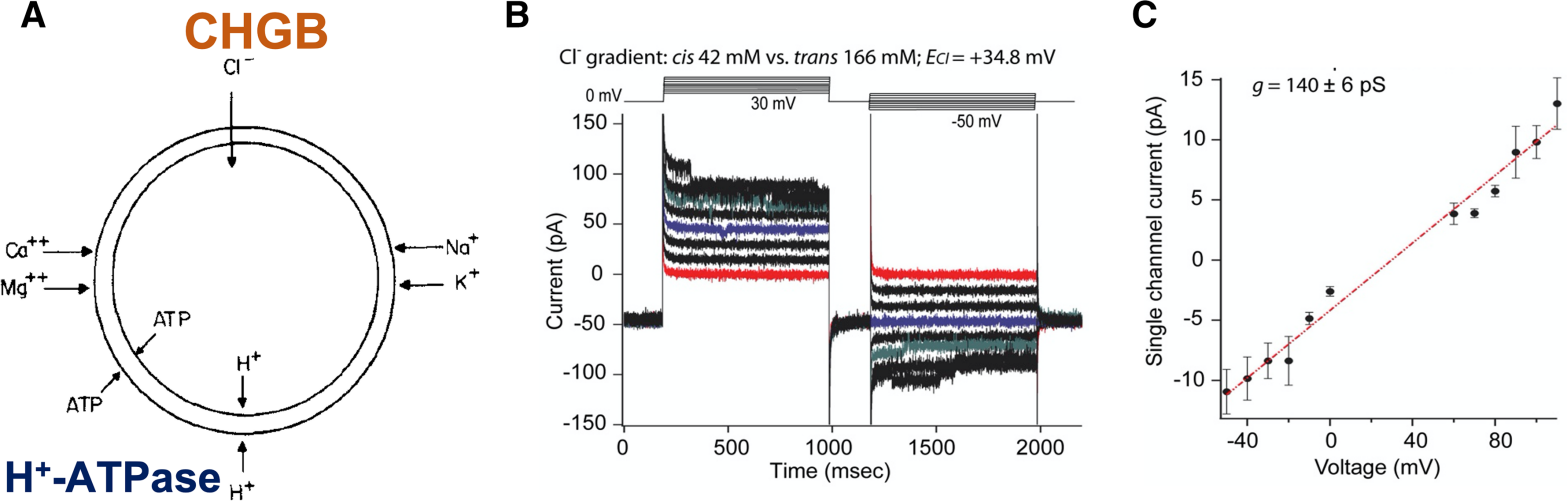
CHGB anion channel and the anion shunt pathway in regulated secretion. (**A**) Chromaffin granules permeate H^+^ in expense of ATP hydrolysis, but not other abundant cations, and they need a Cl^−^ conductance for proper acidification by nullifying positive charges from proton translocation. The CHGB channel is well positioned and good fit to be a critical part, if not the sole one, of this long-missing anion shunt pathway [2]. Adapted from ref. [3] with permissions under the PubMed open access policies. (**B**) Purified recombinant murine CHGB (mCHGB) reconstitutes anion channels in planar lipid bilayers. The presence of Ca^2+^ or Mg^2+^ leads to inactivation at high polarization potentials. (**C**) Single channel currents of mCHGB as a function of transmembrane potential (Vm), giving rise to an average single channel conductance of 140 pS (42/166 mM KCl). Panels **B** and **C** were from ref. [4] with permission under the PubMed open access policies.

An anion shunting conductance in regulated secretory pathways was first proposed in late 1970s when it was observed that the chromaffin granules use an H^+^-ATPase to pump protons into the secretory granules and need fast Cl^−^ influx to neutralize positive charges so that granule acidification can happen quickly (Figure 2A) [3,13,14]. Given the average size of secretory granules (300 nm), it only needs ∼85 free H^+^ to keep pH inside granule at 5.0, and it takes ∼1000 protons to set up a +55 mV potential inside. Because hydrolysis of one ATP by the pump drives 3–4 H^+^ ions to translocate, a small positive potential (<20 mV) in the luminal side is sufficient to halt H^+^-pumping across one pH unit completely. But, if we assume an intragranular protein concentration of ∼200 mg/ml in a dense core secretory granule (DCSG) and 30% of the residues in these proteins are glutamates or aspartates, it will take ∼2 million H^+^ ions to protonate half of these acidic residues and shift intragranular pH significantly. It means that the fast Cl^−^ shunting is a must for the H^+^ pumping to continue steadily and for the granule acidification to happen reliably in a timely fashion. Otherwise, absence of Cl^−^ shunting will delay normal luminal acidification and slow down or even diminish cargo maturation inside a major fraction, if not all, of secretory granules. Granule maturation is important for proper processing of peptide hormones and secretory proteins inside the granules, or for loading of small molecule compounds, such as catecholamines, dopamine, etc., to the luminal side [78–81]. Our recent findings suggest that the CHGB anion channel serves a good candidate as a key component for the long-missing anion shunt conductance [2,4] (Figure 2B,C).

Chromogranins, including CHGA, CHGB (SgI), CHGC (SgII) and CHGD (SgIII), as well as the other five known members of the granin superfamily are obligate granular proteins and function in the regulated secretory pathways in exocrine, endocrine, neuronal, and stem cells [82]. They are present in every tissue or organ of a human body [82]. The CHGA and CHGB were the first to be purified as heat-stable fractions from homogenized tissues, and their genetic identity was revealed in 1980s [83,84]. Since then, nearly all published work in literature was on the soluble forms of the granin proteins. Before our identification of the CHGB channel function [4], studies of two groups suggested that the CHGB may bind to lipid vesicles or stay as a ‘tightly membrane-bound’ form on the cell surface after stimulated granule release [66,85–87]. The ‘tightly-membrane-bound form' could not be dissociated by harsh treatment of NaHCO_3_ at a higher, basic pH, which was regarded as a good indicator of bilayer-spanning transmembrane proteins. In 2018, my group reported that recombinant murine CHGB (mCHGB) of nearly 100% purity became well integrated in membrane after reconstituted into lipid vesicles [4]. More surprisingly, the mCHGB alone suffices to reconstitute a highly selective chloride channel that favors F^−^ and Cl^−^ over Br^−^, I^−^, NO_3_^−^ and other anions quite significantly. The channel has a large conductance (∼140 pS with ∼170 mM Cl^−^; Figure 2C) with a permeation ratio of P_Cl_/P_K_ > 130 : 1, and stays open most of time when *Vm* is within [−50,50] mV. The CHGB channel starts to inactivate when mM divalent cations are present and Vm is higher than 60 or lower than −60 mV. The channel is blocked by DIDS with a K_d_ of ∼450 nM, which is 200–300 fold higher than the reported DIDS sensitivity of the CLC-family proteins, bestrophin proteins, and other known Cl^−^ channels [43,71,88]. Its single channel conductance shows strong sensitivity to Cl^−^ as expected. The high P_Cl_/P_Br_ (∼25 : 1) or P_Cl_/P_I_ is very peculiar [2], probably by following the relative scale of dehydration energy more closely than other anion channels, and makes the CHGB channel much more selective than all other known anion channels except the Fluc channel [57]. The latter selects F^−^ over Cl^−^ and other anions, but is not present in vertebrates [57,58]. The physical laws require that the CHGB channels must span the two leaflets of a membrane bilayer in order to conduct anions in a diffusion-limited fashion [7].

CLC-3 appears not an ideal candidate for the anion-shunting conductance, although it is probably an important modulator of the granule release [89]. Around 2008, ClC-3 was assessed as a possible Cl^−^ loader in regulated secretion with conflicting data from different groups [90–93]. The main discrepancies are in several layers. *First,* instead of a channel as originally thought, CLC-3 is a 2Cl^−^/H^+^ exchanger with fS conductance per functional unit [94]. High-resolution cryo-EM studies suggest a kinetic barrier for the two-gate process of the transporter [95]. *Second,* ClC-3 delivered to the cell surface after overexpression shows strict outward rectification, which should prevent Cl^−^ flow into the granules (inward current) [94,96]. *Third,* ClC-3's physical presence in secretory granules bears uncertainty due to ambiguity in specificity of antibodies used for immuno-histochemistry and low-resolution imaging in immunofluorescence microscopy. Antibodies verified in cells from knockout mice reported CLC-3's absence (or below detection limit) in insulin-secretory granules [97], contrasting with two other studies [91,92]. A more recent study reported that all commercial ClC-3 antibodies failed to detect a ClC-3 splicing variant, ClC-3c, in secretory granules, but a customized antibody by the group did [89], which may need further examination by high-pressure freezing and immuno-electron microscopy or by introducing specific tags to ClC-3 gene by genome-editing [98]. *Fourth,* ClC-3's roles in granule exocytosis showed significant variations in experimental data by different groups. Two earlier studies showed that CLC-3 knockout abolished almost (>85%) all granule-release-triggered increase in membrane capacitance [91,92]. But the recent study showed a clear increase in membrane capacitance of granule exocytosis in chromaffin cells of young mice, but only a minor decrease (<25%) in granule-fusion related capacitance in adult cells [89], casting shadow on the earlier conclusion that CLC-3 is a licensing factor for granule release [91,92]. *Fifth*, a 250 pS Cl^−^ channel assigned to secretory granules bears uncertainty due to potential biochemical contaminants [99,100]. Recently, it was found that VAMP-3 antibody-based affinity purification may overcome most, if not all, of technical issues from unknown contaminants, and could be used to further evaluate this unknown Cl^−^ channel [101]. At the current stage, it is safe to say that these past studies as well as the channel function of CHGB do not exclude, but instead, support more consistently a modulatory role of CLC-3 in regulated secretion. In contrast, the CHGB channels are well positioned to contribute to Cl^−^ shunting [2,4,102].

The role of a cation counterflow against H^+^-translocation in secretory granule faces uncertainty (Figure 1B). Although multiple cation channels were reported in lysosomes and the fluxes of K^+^ or Na^+^ contribute to the lysosomal acidification in the absence of Cl^−^ flow [99,103,104], the chromaffin granules were first found to be impermeant to Na^+^, K^+^, Mg^2+^ or Ca^2+^ [13]. Later, K^+^ channels were identified in isolated zymogen and chromaffin granules [99,100], which suffered from the same biochemical uncertainty due to unknown contaminants. Affinity purification of secretory granules or localization of the expression products from genomically edited genes may be used to resolve this ambiguity more reliably in the future.

## CHGB channels distribute among intracellular membranes and affect organellar anions

**Figure 3.**
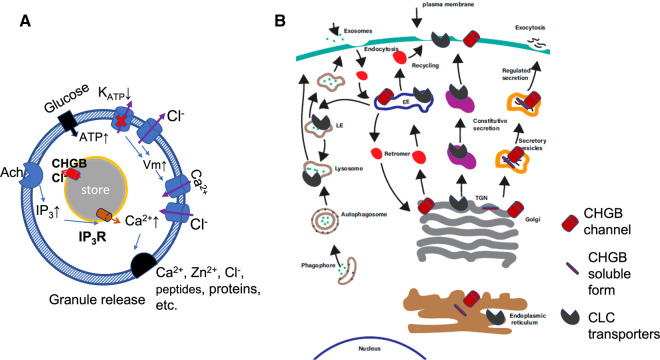
CHGB channel functions in granule release and its distribution among endomembranes and membrane trafficking pathways in a secretory cell. (**A**) In a beta-cell, the glucose and Ach signaling lead to the influx of Ca^2+^ from outside or the release of Ca^2+^ from the insulin-secretory granules. CHGB channels are in both the granular membranes and the plasma membranes and may participate in both pathways. (**B**) CHGB channels go through the endomembrane system, be taken from the cell surface and distributed to the endosomal compartments. CLC transporter proteins are not sorted into the regulated secretory pathway. EE: early endosomes; LE: late endosomes; ERC: endosomal recycling compartments; TGN: trans-Golgi network. Panel **B** was adapted from ref. [1] with permissions following the PubMed Open Access policies.

In a neuroendocrine cell, translation of a CHGB mRNA is initiated in the cytosol before the nascent protein goes through the ER and Golgi apparatus, during which the signal peptide is cleaved, post-translational modifications happen, and protein folding takes place so that only properly folded and maturated CHGB leaves ER and is utilized in the Golgi apparatus for sorting various proteins and small molecules into the regulated secretory pathway (Figure 3) [105]. Because of the dimorphic nature of CHGB, namely existing in both soluble and membrane-integral states, it is expected that a significant fraction of the nascent CHGB is inserted in the membranes of ER and Golgi, and molecular chaperones like HSP70 and HSP90 may help CHGB's folding and dimerization and/or oligomerization. After the biogenesis of secretory granules at the *trans* Golgi network (TGN), whereby the oligomeric CHGB and other granin-aggregates, such as amyloid-like polymerization of cargo components [106,107], CHGA/CHGB oligomerization, membrane-binding-induced SgIII oligomerization [108,109] and/or phase-separation-triggered condensation of SgII (CHGC) [110], etc., may facilitate granule biogenesis in varying degrees, the CHGB protein becomes concentrated in immature secretory granules. During the maturation of secretory granules, proteolysis of the CHGB soluble form gives rise to CHGB-related peptides [111–116], whereas its ‘tightly membrane-bound form' is protected by membranes and remains full-length throughout (Figure 3B). After granule release, the membrane-integrated CHGB channels stay on the cell surface for a short while (e.g. 2–4 h in PC-12 cells [66]) before being taken up via endocytosis. After going through the endosomal compartments, the CHGB-integrated granule membranes are expected to be recycled back to *trans*-Golgi cisternae or the TGN via the endosome recycling compartment (ERC) or lysosome-related organelles (LROs), or even directly fuse with immature secretory granules [1,79,117,118]. A fraction of granular membrane-integrated proteins (including CHGB) may be delivered to lysosomal compartments for degradation. This complicated lifecycle of the CHGB membrane-bound form makes it inevitable that a certain level of CHGB anion channels probably exist in the ER, the Golgi apparatus including the TGN, the plasma membrane, the endosomal compartments, the ERCs, and even some lysosomes, LROs, or autophagosomal compartments [117–119]. Without highly specific blockers to keep them shut, the large conductances of CHGB channels will likely make these compartments permeable to Cl^−^, and help cations in these membrane-enclosed spaces to move in and out quickly and pH control to be achieved readily. The net results are somewhat elevated Cl^−^ concentrations in at least some of these compartments, which can serve as a buffer stock for intracellular Cl^−^ homeostasis (Figure 3B).

## CHGB channels in the plasma membrane and regulation of cytosolic [Cl^−^]

Pancreatic beta-cells are a type of neuroendocrine cells with a high expression level of CHGB protein [78,120]. From the above discussions of anion balance by Cl^−^ loaders and extruders (Figure 1), it is expected that when dozens of CHGB channels (say 25) are on the cell surface, they will generate a huge Cl^−^ conductance (e.g. a total of 3500 pS in the resting condition), allow the passing of millions to billions of anions quickly (e.g. ∼1.0 × 10^9^ Cl^−^ ions moving down 50 mV with 3500 pS in one second), and help bring quickly the intracellular Cl^−^ concentration close to a steady-state level so that the reversal potential (E_Cl_) for Cl^−^ is close to the transmembrane potential (Vm), whereby the net Cl flux is minimized. This steady-state level suggests that for a beta-cell with an intracellular Cl^−^ concentration of ∼34 mM, its resting Vm is expected to be at ∼−40 mV due to the CHGB conductance on the cell surface, which is very close to what was measured from the primary beta-cells in the resting condition [18,121].

During granule release, more CHGB channels will be delivered to the cell surface. Action potentials are triggered by *Vm* depolarization after the closure of K_ATP_ channels and activities of voltage-gated Na^+^ channels and L-type voltage-gated Ca^2+^ channels (Figure 3A) [122]. The influx of Ca^2+^ leads to the fusion of the granules with the plasma membranes. After granule release, if we still use a pancreatic beta-cell as an example, the number of CHGB channels on the cell surface may be 10–20 folds higher than that under the resting condition, *a.k.a.* ∼400 CHGB channels/per cell [2]. The large CHGB conductance will drive *Vm* repolarization due to a large Cl^−^ influx. This will probably shorten the action potential duration in the beta cells [121], and may increase the chance of burst firing. From the angle of Cl^−^ homeostasis, the large CHGB conductance will favor stabilization of transmembrane potential close to the reversal potential of Cl^−^, instead of being dominated by the K^+^ conductance (including both K_ATP_ and Kv channels) and thus set by the reversal potential of K^+^, which is determined mainly by Na-K-ATPase (Na-pump), NKCC, KCC, etc. Up-regulating Cl^−^ transporters (NCCs, NKCCs and AEs, etc.) that load the Cl^−^ will increase the resting intracellular Cl^−^ concentration. The CHGB channel on the cell surface is therefore an important factor in the controlled batch-release of insulin-secretory granules from multiple electrically coupled beta-cells, which may be related to the reported genetic associations of *Chgb* loci with type-2 diabetes in the human population worldwide [67,123].

After a short period (e.g. 2–4 h in PC-12 cells), the CHGB channels will be removed from the cell surface so that the surface Cl^−^ conductance will be reduced to the resting level [66]. In the *in vivo* conditions, beta-cells may undergo multiple cycles of granule release every day such that there are likely a significant number of CHGB channels on the cell surface for an extended period of time, which expectedly will make the intracellular Cl^−^ level higher and the transmembrane potential to be less negative than other cells (closer to the threshold Vm), enhancing the likelihood for action potential firing among the beta-cells [121] and making it easier to release granules (a positive feedback).

An additional advantage of the elevated [Cl^−^]_i_ in the beta cells is that they will have sufficient cytosolic Cl^−^ for anion shunting to work efficiently in individual secretory granules. Each beta-cell has tens of thousands of secretory granules [124], which in total would need ∼2.0 × 10^10^ Cl^−^ ions for proper acidification to happen. The total amount of cytosolic Cl^−^ in a beta cell is estimated to be 9.0 × 10^10^ (∼35 mM), sufficient to satisfy the needs of the secretory granules. A prediction from this comparison is that the shortage of Cl^−^ loaders (e.g. NKCC) in the beta-cells should lead to partial impairment of granule acidification, and in sequence, slow down normal insulin secretion, which has been reported before [125]. Similarly, inhibition of Cl^−^ extruders (e.g. KCC2) in the beta cells up-regulates intracellular [Cl^−^] and stimulates both basal and glucose-stimulated insulin secretion [31].

## CHGB channels in ER, Golgi apparatus and dense core granules

When CHGB is expressed continuously to replenish the regulated secretory pathway, there will be a fraction of CHGB proteins that are inserted in the ER membranes after being folded properly. Presumably, they reconstitute conducting channels and allow Cl^−^ ions to cross the ER membrane. The anion conductance in the ER membranes used to be assigned to the CLIC channel [126,127], which is a misname because the CLIC channels have been showed to be non-selective or even more likely cation-selective. When the ER is loaded with Ca^2+^ by Ca-ATPase, it requires either Cl^−^ ions to move in, or cations, such as K^+^ to move out through the trimeric intracellular cation (TRIC) channel or other cation channels [74,128]. Because voltage-gated channels tend to inactivate quickly near zero transmembrane potential, we don't expect them to be very efficient in balancing charges of the ER compartment. The TRIC channels become nonconductive when Ca^2+^ is high (*K_i_* ∼ 0.4 mM), and only are active when the Ca^2+^ level is significantly low [74]. The CHGB conductance is not blocked by Ca^2+^, and can function effectively in the presence of 1–5 mM Ca^2+^, which may be helpful for sustained pumping of Ca^2+^ to maintain a free Ca^2+^ level of 1–2 mM in the ER lumen (in CHGB+ cells). The ER membranes have large surface areas. In a typical ER tubular structure of 150–200 nm in diameter, ∼2000 Ca^2+^ ions are sufficient to charge a 1.0 micron-long tubule to +100 mV (relative to the cytosol), which can barely increase the Ca^2+^ concentration by 0.1 mM, but is sufficient to shut down the Ca^2+^-ATPase. It is hence beneficial to have a Cl^−^ conductance in the ER membranes that functions in the presence of ∼1.0 mM Ca^2+^ so that Ca^2+^ loading can continue to a higher level when the TRIC channels are significantly inhibited. In cells expressing no CHGB, other cation or anion channels may be recruited to serve the same purposes. Similarly, for the Ca^2+^ release by ion channels, such as ryanodine and IP_3_-receptors [129,130], the fast release requires a counter-flow of cations or a parallel flux of anions (Cl^−^; Figure 3). The CHGB channels in ER membranes, if present, obviously can support this function so that the Ca^2+^ release (as spikes or waves) can happen transiently within ms duration. The same may happen in nuclei if CHGB is present in the nuclear membranes, not limited to the inner nuclear membrane [131,132].

CHGB channels should play similar roles in the Golgi apparatus as in the ER. The individual stacks of Golgi cisternal membranes need Cl^−^ conductances or cation channels for Ca^2+^-loading or -release [133,134]. CHGB can serve as the anion channels in the CHGB+ cells. In the IP_3_R-containing dense-core secretory granules (DCSGs), right before secretory granule release, the signaling (via acetylcholine) from the cell surface (Figure 3A) may lead to activation of phospholipase C (PLC), which produces IP_3_ to trigger calcium release from the granules. An accompanying efflux of Cl^−^ through the CHGB channels or an influx of cations (e.g. maybe via TPC1/2 channels [135]? or other cation channels) is required for the fast Ca^2+^ release [136]. The large conductance of CHGB channels is advantageous in adding another route to fulfill such a role so that Ca^2+^ release from the granular stores can occur very quickly.

## CHGB channels for Cl^−^ accumulation in endosomal and endolysosomal compartments

Similar to Cl^−^ accumulation in secretory granules (Figure 2A), it is expected that when the CHGB channels are retrieved from the cell surface into the endosomal compartments, weak acidification of these compartments driven by proton-ATPase will drive the Cl^−^ flux through the CHGB channels, and lead to Cl^−^ accumulation (Figure 3B). In a conventional view, certainly in cells without CHGB expression, 2Cl^−^/H^+^ exchangers like ClC-3, -5, or -7 are used to cancel out 33% of the work by the proton-ATPase and achieve charge neutralization by a net translocation of 2H^+^ and 2Cl^−^ at the expense of 3 ATP [34,103,104,137], which is a wasteful way in comparison with the CHGB conductance, but allows a fine control of the pumping cycles and Cl^−^ accumulation. It is expected that the slow acidification in the endosomal compartments does not ask for fast flux of anions and the protein concentration inside these compartments are much less than that of the DCSGs so that either CLC family exchangers or the CHGB channels can serve the needs of generating a Cl^−^ influx well.

In the endolysosomal compartments, luminal acidification is even stronger than the secretory granules (pH < 5.0; Figure 3B) such that the high concentration of proteins inside, especially various enzymes, requires the transportation of a large number of protons for protonation of acidic residues. The presence of CHGB channels may still be functional in a pH lower than that of the mature secretory granules (∼pH 5.5), and allow fast flux of sufficient Cl^−^ ions for charge balance (millions or more for each lysosomal compartment that is ∼100 nm in diameter). In both endolysosomal and lysosomal compartments, counterflows of K^+^ and Na^+^ against H^+^ translocation may contribute to charge balance as well as the CLC antiporters (ClC-3,-5, or -7) [103].

## CHGB and cellular anion homeostasis in regulated secretory cells

In CHGB+ cells, the need for Cl^−^ homeostasis is satisfied by Cl^−^ loaders and extruders as usual (Figure 1A). For a cell that releases secretory granules, every batch release of granules dumps out a large amount of Cl^−^ ions (millions to billions per release, say ∼50 readily releasable granules) [124], which need to be compensated for by Cl^−^ loaders on the cell surface [18]. Usually, the known families of small conductance Cl^−^ channels on the cell surfaces (without overexpression) may not be able to load so many anions quickly, but the large-conductance CHGB channels delivered to the cell surface in synchrony with granule release can do it well. During this process, the exquisite selectivity of the CHGB channel prevents other anions from being concentrated into secretory granules and being lost to the extracellular side and on the cell surface, keeps other anions, such as HCO_3_^−^ and NO_3_^−^, from going into the cell. After this task is done, the intact CHGB channels are retrieved back from the cell surface. If the quick compensation through the CHGB channels is not enough, a cell may lose too many anions after repeated release of thousands of granules in a short period of time, and become hypotonic and be swollen. If so, VRACs (volume regulated anion channels) will be recruited to adjust and restore cell volumes [43,59,60,62,138–140]. Even in the presence of the transient roles of the CHGB channels on the cell surface, the main drivers for sustained Cl^−^ loading are still the active transporters, such as NCCs, NKCCs and AEs.

Because CHGB channels have high open probability in the Vm range of [−50,50] mV [4], their large conductance on the cell surface will shift the transmembrane potential towards the Nernst potential of Cl^−^, which might be fairly costly for a cell's ability in maintaining minimal or no chloride conduction in the resting steady-state when necessary. If so, it is important to remove all or almost all CHGB channels from the cell surface or find a way to keep the CHGB channels shut after their delivery with secretory granules. Similar to the synaptic vesicle membranes after fusion, the CHGB and other granular membrane components may stay clustered together on the cell surface [141,142], and are presumably taken up via the clathrin-mediated endocytosis [81,142].

## Conclusions

The CHGB anion channels, if being open as observed for recombinant channels, should add significant chloride conductances into all membranes they reside in. In intracellular membranes, they facilitate cation loading or release via charge neutralization and support chloride accumulation in calcium stores in different intracellular compartments. Their high selectivity of Cl^−^ over almost all other main intracellular anions abrogates unintended translocation of the latter group to the luminal side and wasteful dumping to the extracellular side after granule release. At the cell surface, transient presence of CHGB channels enables the utilization of their large conductance to achieve fast flow of Cl^−^ across the plasma membrane and facilitate replenishment of Cl^−^ loss during repetitive release of a large number of secretory granules in a short period of time. The Cl^−^ homeostasis is thus supported by CHGB channels in cells expressing them, especially in regulated secretory pathways, which reveals a critical aspect of CHGB intracellular functions and may be associated with CHGB-related human diseases.

## Perspectives

- CHGB is present in human cells that utilize regulated secretion to send signals to the outside. CHGB-mediated Cl^−^ homeostasis is thus of critical importance to signal release, cation storage and release, and cell volume control in CHGB+ cells.
- The current framework of cellular Cl^−^ homeostasis includes low-selectivity Cl^−^ channels with small conductance, transporters of high turnover rates, or cation channels in some cases. The highly selective, large-conductance CHGB channels are advantageous to overcome some critical limitations in the current paradigm.
- As the CHGB anion channel is new to the field, many questions remain open on its transmembrane topology in forming the ion-conducting pore, its roles in adjusting [Cl^−^]_i_, Cl^−^ accumulation in organellar space, and cell volume control in normal CHGB+ cells, other cells that transiently become CHGB+, and neuroendocrine transforms of cancer cells or other immortalized cells. How CHGB channels and other known Cl^−^ channels or transporters are coordinated in a cell to achieve real-time control of Cl^−^ homeostasis under different phases of a cell's life is yet another mystery that awaits further studies.

## Open Access

Open access for this article was enabled by the participation of University of Florida in an all-inclusive *Read & Publish* agreement with Portland Press and the Biochemical Society under a transformative agreement with Individual.

## Abbreviations

Ach: acetylcholine
AE: anion exchanger (HCO_3_^−^-Cl^−^ antiporter)
CHGA: chromogranin A, which used to be called CgA in literature
CHGB: chromogranin B which was also called CgB or SgI in brevity in literature
CHGC: chromogranin C, which was also called CgC or SgII in literature
CHGD: chromogranin D, which was also called CgD or SgIII in literature
Cl^−^: chloride ion
[Cl^−^]_i_: intracellular chloride concentration
CLC: voltage-gated chloride channels or electrogenic 2Cl^−^/H^+^ transporters (CLC3–7)
CNS: central nervous system
DCSG: dense-core secretory granules
ER: endoplasmic reticulum
ERC: endosomal recycling compartment
GABA: gamma-aminobutyric acid or 4-aminobutanoic acid
KCC: K^+^-Cl^−^ cotransporter
NCC: Na^+^-Cl^−^ cotransporter
NKCC: Na^+^-K^+^-2Cl^−^ cotransporter
SLC: solute carrier family
TGN: *trans*-Golgi network
*Vm*: transmembrane electrostatic potential
*Erev*: reversal transmembrane potential
*E_Cl_*: Nernst potential of Cl^−^

## References

[1] O'Sullivan, M.J. and Lindsay, A.J. (2020) The endosomal recycling pathway-at the crossroads of the cell. Int. J. Mol. Sci. 21, 6074 10.3390/ijms21176074 32842549 PMC7503921

[2] Yadav, G., Wang, H., Ouwendijk, J., Annamalai, M., Cross, S., Wang, Q. et al. (2019) Membrane insertion of chromogranin B for granule maturation in regulated secretion. BioRxiv, 1–61. 10.1101/2019.12.28.890053

[3] Johnson, R.G., Carty, S.E. and Scarpa, A. (1985) Coupling of H^+^ gradients to catecholamine transport in chromaffin granules. Ann. N. Y. Acad. Sci. 456, 254–267 10.1111/j.1749-6632.1985.tb14874.x 2868684

[4] Yadav, G., Zheng, H., Yang, Q., Douma, L.G., Bloom, L.B. and Jiang, Q.-X. (2018) Secretory granule protein chromogranin B (CHGB) forms an anion channel in membrane. Life Sci. Alliance 1, e201800139 10.26508/lsa.201800139 30456382 PMC6238609

[5] Alberts, B., Johnson, A., Lewis, J., Raff, M., Roberts, K. and Walter, P. (2007) Molecular Biology of the Cell, 5th ed, Galand Science, New York

[6] Iwasa, J. and Marshall, W. (2015) Karp's Cell and Molecular Biology, Wiley, USA

[7] Hille, B. (2001) Ion Channels of Excitable Membranes, 3rd ed, Sinauer Associates, Inc, Sunderland, MA, USA

[8] Hodgkin, A.F. and Huxley, A.F. (1952) A quantitative description of membrane current and its application to conduction and excitation in nerve. J. Physiol. 117, 500–544 267 10.1113/jphysiol.1952.sp004764 12991237 PMC1392413

[9] Eccles, J.C. (1951) Interpretation of action potentials evoked in the cerebral cortex. Electroencephalogr. Clin. Neurophysiol. 3, 449–464 10.1016/0013-4694(51)90033-8 14887631

[10] Sudhof, T.C. and Jahn, R. (1991) Proteins of synaptic vesicles involved in exocytosis and membrane recycling. Neuron 6, 665–677 10.1016/0896-6273(91)90165-v 1673848

[11] Jahn, R. and Sudhof, T.C. (1994) Synaptic vesicles and exocytosis. Annu. Rev. Neurosci. 17, 219–246 10.1146/annurev.ne.17.030194.001251 8210174

[12] Sudhof, T.C. and Rothman, J.E. (2009) Membrane fusion: grappling with SNARE and SM proteins. Science 323, 474–477 10.1126/science.1161748 19164740 PMC3736821

[13] Johnson, R.G. and Scarpa, A. (1976) Ion permeability of isolated chromaffin granules. J. Gen. Physiol. 68, 601–631 10.1085/jgp.68.6.601 11272 PMC2228447

[14] Johnson, R.G. and Scarpa, A. (1976) Internal pH of isolated chromaffin vesicles. J. Biol. Chem. 251, 2189–2191 PMID:5444

[15] Rahmati, N., Hoebeek, F.E., Peter, S. and De Zeeuw, C.I. (2018) Chloride homeostasis in neurons with special emphasis on the olivocerebellar system: differential roles for transporters and channels. Front. Cell Neurosci. 12, 101 10.3389/fncel.2018.00101 29765304 PMC5938380

[16] Ghit, A., Assal, D., Al-Shami, A.S. and Hussein, D.E.E. (2021) GABAA receptors: structure, function, pharmacology, and related disorders. J. Genet. Eng. Biotechnol. 19, 123 10.1186/s43141-021-00224-0 34417930 PMC8380214

[17] Kim, S., Ma, L., Unruh, J., McKinney, S. and Yu, C.R. (2015) Intracellular chloride concentration of the mouse vomeronasal neuron. BMC Neurosci. 16, 90 10.1186/s12868-015-0230-y 26667019 PMC4678706

[18] Di Fulvio, M. and Aguilar-Bryan, L. (2019) Chloride transporters and channels in beta-cell physiology: revisiting a 40-year-old model. Biochem. Soc. Trans. 47, 1843–1855 10.1042/BST20190513 31697318 PMC6925527

[19] Wilke, B.U., Kummer, K.K., Leitner, M.G. and Kress, M. (2020) Chloride: the underrated ion in nociceptors. Front. Neurosci. 14, 287 10.3389/fnins.2020.00287 32322187 PMC7158864

[20] Saint-Criq, V., Guequen, A., Philp, A.R., Villanueva, S., Apablaza, T., Fernandez-Moncada, I. et al. (2022) Inhibition of the sodium-dependent HCO(3)(-) transporter SLC4A4, produces a cystic fibrosis-like airway disease phenotype. eLife 11, e75871 10.7554/eLife.75871 35635440 PMC9173743

[21] Devor, D.C., Singh, A.K., Lambert, L.C., DeLuca, A., Frizzell, R.A. and Bridges, R.J. (1999) Bicarbonate and chloride secretion in calu-3 human airway epithelial cells. J. Gen. Physiol. 113, 743–760 10.1085/jgp.113.5.743 10228185 PMC2222914

[22] Jennings, M.L. (2021) Cell physiology and molecular mechanism of anion transport by erythrocyte band 3/AE1. Am. J. Physiol. Cell Physiol. 321, C1028–C1C59 10.1152/ajpcell.00275.2021 34669510 PMC8714990

[23] Lurin, C., Guclu, J., Cheniclet, C., Carde, J.P., Barbier-Brygoo, H. and Maurel, C. (2000) CLC-Nt1, a putative chloride channel protein of tobacco, co-localizes with mitochondrial membrane markers. Biochem. J. 348, 291–295 PMID:10816421 PMC1221065

[24] De Angeli, A., Monachello, D., Ephritikhine, G., Frachisse, J.M., Thomine, S., Gambale, F. et al. (2006) The nitrate/proton antiporter AtCLCa mediates nitrate accumulation in plant vacuoles. Nature 442, 939–942 10.1038/nature05013 16878138

[25] De Angeli, A., Monachello, D., Ephritikhine, G., Frachisse, J.M., Thomine, S., Gambale, F. et al. (2009) Review. CLC-mediated anion transport in plant cells. Philos. Trans. R. Soc. Lond. B Biol. Sci. 364, 195–201 10.1098/rstb.2008.0128 18957376 PMC2674092

[26] Monachello, D., Allot, M., Oliva, S., Krapp, A., Daniel-Vedele, F., Barbier-Brygoo, H. et al. (2009) Two anion transporters atClCa and atClCe fulfil interconnecting but not redundant roles in nitrate assimilation pathways. New Phytol. 183, 88–94 10.1111/j.1469-8137.2009.02837.x 19402883

[27] Wege, S., Jossier, M., Filleur, S., Thomine, S., Barbier-Brygoo, H., Gambale, F. et al. (2010) The proline 160 in the selectivity filter of the arabidopsis NO_3_^−^/H^+^ exchanger AtCLCa is essential for nitrate accumulation in planta. Plant J. 63, 861–869 10.1111/j.1365-313X.2010.04288.x 20598093

[28] Orlov, S.N., Koltsova, S.V., Kapilevich, L.V., Gusakova, S.V. and Dulin, N.O. (2015) NKCC1 and NKCC2: The pathogenetic role of cation-chloride cotransporters in hypertension. Genes Dis. 2, 186–196 10.1016/j.gendis.2015.02.007 26114157 PMC4477834

[29] Gamba, G. (2005) Molecular physiology and pathophysiology of electroneutral cation-chloride cotransporters. Physiol. Rev. 85, 423–493 10.1152/physrev.00011.2004 15788703

[30] Rojas-Vega, L. and Gamba, G. (2016) Mini-review: regulation of the renal NaCl cotransporter by hormones. Am. J. Physiol. Renal Physiol. 310, F10–F14 10.1152/ajprenal.00354.2015 26511649

[31] Kursan, S., McMillen, T.S., Beesetty, P., Dias-Junior, E., Almutairi, M.M., Sajib, A.A. et al. (2017) The neuronal K^+^Cl^−^ co-transporter 2 (Slc12a5) modulates insulin secretion. Sci. Rep. 7, 1732 10.1038/s41598-017-01814-0 28496181 PMC5431760

[32] Jentsch, T.J. (2015) Discovery of CLC transport proteins: cloning, structure, function and pathophysiology. J. Physiol. 593, 4091–4109 10.1113/JP270043 25590607 PMC4594286

[33] Stolting, G., Fischer, M. and Fahlke, C. (2014) CLC channel function and dysfunction in health and disease. Front. Physiol. 5, 378 10.3389/fphys.2014.00378 25339907 PMC4188032

[34] Jentsch, T.J. and Pusch, M. (2018) CLC chloride channels and transporters: structure, function, physiology, and disease. Physiol. Rev. 98, 1493–1590 10.1152/physrev.00047.2017 29845874

[35] Lopes-Pacheco, M. (2019) CFTR modulators: the changing face of cystic fibrosis in the era of precision medicine. Front. Pharmacol. 10, 1662 10.3389/fphar.2019.01662 32153386 PMC7046560

[36] Kerem, B., Rommens, J.M., Buchanan, J.A., Markiewicz, D., Cox, T.K., Chakravarti, A. et al. (1989) Identification of the cystic fibrosis gene: genetic analysis. Science 245, 1073–1080 10.1126/science.2570460 2570460

[37] Lobet, S. and Dutzler, R. (2006) Ion-binding properties of the ClC chloride selectivity filter. EMBO J. 25, 24–33 10.1038/sj.emboj.7600909 16341087 PMC1356352

[38] Dutzler, R., Campbell, E.B. and MacKinnon, R. (2003) Gating the selectivity filter in ClC chloride channels. Science 300, 108–112 10.1126/science.1082708 12649487

[39] Dutzler, R., Campbell, E.B., Cadene, M., Chait, B.T. and MacKinnon, R. (2002) X-ray structure of a ClC chloride channel at 3.0 A reveals the molecular basis of anion selectivity. Nature 415, 287–294 10.1038/415287a 11796999

[40] Huang, W.C., Xiao, S., Huang, F., Harfe, B.D., Jan, Y.N. and Jan, L.Y. (2012) Calcium-activated chloride channels (CaCCs) regulate action potential and synaptic response in hippocampal neurons. Neuron 74, 179–192 10.1016/j.neuron.2012.01.033 22500639 PMC3329964

[41] Huang, F., Wong, X. and Jan, L.Y. (2012) International union of basic and clinical pharmacology. LXXXV: calcium-activated chloride channels. Pharmacol. Rev. 64, 1–15 10.1124/pr.111.005009 22090471 PMC3250081

[42] Cruz-Rangel, S., De Jesus-Perez, J.J., Contreras-Vite, J.A., Perez-Cornejo, P., Hartzell, H.C. and Arreola, J. (2015) Gating modes of calcium-activated chloride channels TMEM16A and TMEM16B. J. Physiol. 593, 5283–5298 10.1113/JP271256 26728431 PMC4704513

[43] Ullrich, F., Blin, S., Lazarow, K., Daubitz, T., von Kries, J.P. and Jentsch, T.J. (2019) Identification of TMEM206 proteins as pore of PAORAC/ASOR acid-sensitive chloride channels. eLife 8, e49187 10.7554/eLife.49187 31318332 PMC6663466

[44] Yang, J., Chen, J., Del Carmen Vitery, M., Osei-Owusu, J., Chu, J., Yu, H. et al. (2019) PAC, an evolutionarily conserved membrane protein, is a proton-activated chloride channel. Science 364, 395–399 10.1126/science.aav9739 31023925 PMC7305803

[45] Neussert, R., Muller, C., Milenkovic, V.M. and Strauss, O. (2010) The presence of bestrophin-1 modulates the Ca^2+^ recruitment from Ca^2+^ stores in the ER. Pflugers Arch. 460, 163–175 10.1007/s00424-010-0840-2 20411394

[46] Yang, T., Liu, Q., Kloss, B., Bruni, R., Kalathur, R.C., Guo, Y. et al. (2014) Structure and selectivity in bestrophin ion channels. Science 346, 355–359 10.1126/science.1259723 25324390 PMC4341822

[47] Vaisey, G., Miller, A.N. and Long, S.B. (2016) Distinct regions that control ion selectivity and calcium-dependent activation in the bestrophin ion channel. Proc. Natl Acad. Sci. U.S.A. 113, E7399–EE408 10.1073/pnas.1614688113 27821745 PMC5127342

[48] Fischmeister, R. and Hartzell, H.C. (2005) Volume sensitivity of the bestrophin family of chloride channels. J. Physiol. 562, 477–491 10.1113/jphysiol.2004.075622 15564283 PMC1665509

[49] O'Halloran, D.M. (2022) Database of glutamate-gated chloride (GluCl) subunits across 125 nematode species: patterns of gene accretion and sequence diversification. G3 (Bethesda) 12, jkab438 10.1093/g3journal/jkab438 35100348 PMC9210312

[50] Hibbs, R.E. and Gouaux, E. (2011) Principles of activation and permeation in an anion-selective Cys-loop receptor. Nature 474, 54–60 10.1038/nature10139 21572436 PMC3160419

[51] Gulacsi, A., Lee, C.R., Sik, A., Viitanen, T., Kaila, K., Tepper, J.M. et al. (2003) Cell type-specific differences in chloride-regulatory mechanisms and GABA(A) receptor-mediated inhibition in rat substantia nigra. J. Neurosci. 23, 8237–8246 10.1523/JNEUROSCI.23-23-08237.2003 12967985 PMC6740695

[52] Du, J., Lu, W., Wu, S., Cheng, Y. and Gouaux, E. (2015) Glycine receptor mechanism elucidated by electron cryo-microscopy. Nature 526, 224–229 10.1038/nature14853 26344198 PMC4659708

[53] Kasaragod, V.B. and Schindelin, H. (2018) Structure-function relationships of glycine and GABAA receptors and their interplay with the scaffolding protein gephyrin. Front. Mol. Neurosci. 11, 317 10.3389/fnmol.2018.00317 30258351 PMC6143783

[54] Huang, X., Shaffer, P.L., Ayube, S., Bregman, H., Chen, H., Lehto, S.G. et al. (2017) Crystal structures of human glycine receptor alpha3 bound to a novel class of analgesic potentiators. Nat. Struct. Mol. Biol. 24, 108–113 10.1038/nsmb.3329 27991902

[55] Stockbridge, R.B., Koide, A., Miller, C. and Koide, S. (2014) Proof of dual-topology architecture of Fluc F-channels with monobody blockers. Nat. Commun. 5, 5120 10.1038/ncomms6120 25290819 PMC4265568

[56] Ji, C., Stockbridge, R.B. and Miller, C. (2014) Bacterial fluoride resistance, Fluc channels, and the weak acid accumulation effect. J. Gen. Physiol. 144, 257–261 10.1085/jgp.201411243 25156118 PMC4144673

[57] Stockbridge, R.B., Robertson, J.L., Kolmakova-Partensky, L. and Miller, C. (2013) A family of fluoride-specific ion channels with dual-topology architecture. eLife 2, e01084 10.7554/eLife.01084 23991286 PMC3755343

[58] Baker, J.L., Sudarsan, N., Weinberg, Z., Roth, A., Stockbridge, R.B. and Breaker, R.R. (2012) Widespread genetic switches and toxicity resistance proteins for fluoride. Science 335, 233–235 10.1126/science.1215063 22194412 PMC4140402

[59] Kefauver, J.M., Saotome, K., Dubin, A.E., Pallesen, J., Cottrell, C.A., Cahalan, S.M. et al. (2018) Structure of the human volume regulated anion channel. eLife 7, e38461 10.7554/eLife.38461 30095067 PMC6086657

[60] Qiu, Z., Dubin, A.E., Mathur, J., Tu, B., Reddy, K., Miraglia, L.J. et al. (2014) SWELL1, a plasma membrane protein, is an essential component of volume-regulated anion channel. Cell 157, 447–458 10.1016/j.cell.2014.03.024 24725410 PMC4023864

[61] Liu, F., Zhang, Z., Csanady, L., Gadsby, D.C. and Chen, J. (2017) Molecular structure of the human CFTR ion channel. Cell 169, 85–95.e8 10.1016/j.cell.2017.02.024 28340353

[62] Deneka, D., Sawicka, M., Lam, A.K.M., Paulino, C. and Dutzler, R. (2018) Structure of a volume-regulated anion channel of the LRRC8 family. Nature 558, 254–259 10.1038/s41586-018-0134-y 29769723

[63] Camara, A.K.S., Zhou, Y., Wen, P.C., Tajkhorshid, E. and Kwok, W.M. (2017) Mitochondrial VDAC1: a key gatekeeper as potential therapeutic target. Front. Physiol. 8, 460 10.3389/fphys.2017.00460 28713289 PMC5491678

[64] Bayrhuber, M., Meins, T., Habeck, M., Becker, S., Giller, K., Villinger, S. et al. (2008) Structure of the human voltage-dependent anion channel. Proc. Natl Acad. Sci. U.S.A. 105, 15370–5 10.1073/pnas.0808115105 18832158 PMC2557026

[65] Parker, M.W. and Feil, S.C. (2005) Pore-forming protein toxins: from structure to function. Prog. Biophys. Mol. Biol. 88, 91–142 10.1016/j.pbiomolbio.2004.01.009 15561302

[66] Pimplikar, S.W. and Huttner, W.B. (1992) Chromogranin B (secretogranin I), a secretory protein of the regulated pathway, is also present in a tightly membrane-associated form in PC12 cells. J. Biol. Chem. 267, 4110–4118 PMID:1740454

[67] Obermuller, S., Calegari, F., King, A., Lindqvist, A., Lundquist, I., Salehi, A. et al. (2010) Defective secretion of islet hormones in chromogranin-B deficient mice. PLoS ONE 5, e8936 10.1371/journal.pone.0008936 20126668 PMC2812483

[68] Diaz-Vera, J., Morales, Y.G., Hernandez-Fernaud, J.R., Camacho, M., Montesinos, M.S., Calegari, F. et al. (2010) Chromogranin B gene ablation reduces the catecholamine cargo and decelerates exocytosis in chromaffin secretory vesicles. J. Neurosci. 30, 950–957 10.1523/JNEUROSCI.2894-09.2010 20089903 PMC6633114

[69] Zhang, K., Biswas, N., Gayen, J.R., Miramontes-Gonzalez, J.P., Hightower, C.M., Mustapic, M. et al. (2014) Chromogranin B: intra- and extra-cellular mechanisms to regulate catecholamine storage and release, in catecholaminergic cells and organisms. J. Neurochem. 129, 48–59 10.1111/jnc.12527 24266713 PMC3992281

[70] Pusch, M., Steinmeyer, K. and Jentsch, T.J. (1994) Low single channel conductance of the major skeletal muscle chloride channel, ClC-1. Biophys. J. 66, 149–152 10.1016/S0006-3495(94)80753-2 8130334 PMC1275674

[71] Chien, L.T., Zhang, Z.R. and Hartzell, H.C. (2006) Single Cl^−^ channels activated by Ca^2+^ in Drosophila S2 cells are mediated by bestrophins. J. Gen. Physiol. 128, 247–259 10.1085/jgp.200609581 16940553 PMC2151570

[72] Suzuki, M., Morita, T. and Iwamoto, T. (2006) Diversity of Cl^−^ channels. Cell. Mol. Life Sci. 63, 12–24 10.1007/s00018-005-5336-4 16314923 PMC2792346

[73] Stolting, G., Teodorescu, G., Begemann, B., Schubert, J., Nabbout, R., Toliat, M.R. et al. (2013) Regulation of ClC-2 gating by intracellular ATP. Pflugers Arch. 465, 1423–1437 10.1007/s00424-013-1286-0 23632988 PMC3778897

[74] Wang, X.H., Su, M., Gao, F., Xie, W., Zeng, Y., Li, D.L. et al. (2019) Structural basis for activity of TRIC counter-ion channels in calcium release. Proc. Natl Acad. Sci. U.S.A. 116, 4238–4243 10.1073/pnas.1817271116 30770441 PMC6410872

[75] Yoo, S.H. (2000) Coupling of the IP3 receptor/Ca^2+^ channel with Ca^2+^ storage proteins chromogranins A and B in secretory granules. Trends Neurosci. 23, 424–428 10.1016/s0166-2236(00)01621-0 10941192

[76] Rekhtman, N. (2022) Lung neuroendocrine neoplasms: recent progress and persistent challenges. Mod. Pathol. 35, 36–50 10.1038/s41379-021-00943-2 34663914 PMC8695375

[77] Puca, L., Vlachostergios, P.J. and Beltran, H. (2019) Neuroendocrine differentiation in prostate cancer: emerging biology, models, and therapies. Cold Spring Harb. Perspect. Med. 9, a030593 10.1101/cshperspect.a030593 29844220 PMC6360865

[78] Omar-Hmeadi, M. and Idevall-Hagren, O. (2021) Insulin granule biogenesis and exocytosis. Cell. Mol. Life Sci. 78, 1957–1970 10.1007/s00018-020-03688-4 33146746 PMC7966131

[79] Ma, C.J., Yang, Y., Kim, T., Chen, C.H., Polevoy, G., Vissa, M. et al. (2020) An early endosome-derived retrograde trafficking pathway promotes secretory granule maturation. J. Cell Biol. 219, e201808017 10.1083/jcb.201808017 32045479 PMC7055004

[80] Kogel, T. and Gerdes, H.H. (2010) Maturation of secretory granules. Results Probl. Cell Differ. 50, 1–20 10.1007/400_2009_31 19888562

[81] Tooze, S.A. and Stinchcombe, J.C. (1992) Biogenesis of secretory granules. Semin. Cell Biol. 3, 357–366 10.1016/1043-4682(92)90021-m 1457778

[82] Bartolomucci, A., Possenti, R., Mahata, S.K., Fischer-Colbrie, R., Loh, Y.P. and Salton, S.R. (2011) The extended granin family: structure, function, and biomedical implications. Endocr. Rev. 32, 755–797 10.1210/er.2010-0027 21862681 PMC3591675

[83] Benedum, U.M., Lamouroux, A., Konecki, D.S., Rosa, P., Hille, A., Baeuerle, P.A. et al. (1987) The primary structure of human secretogranin I (chromogranin B): comparison with chromogranin A reveals homologous terminal domains and a large intervening variable region. EMBO J. 6, 1203–1211 10.1002/j.1460-2075.1987.tb02355.x 3608978 PMC553920

[84] Benedum, U.M., Baeuerle, P.A., Konecki, D.S., Frank, R., Powell, J., Mallet, J. et al. (1986) The primary structure of bovine chromogranin A: a representative of a class of acidic secretory proteins common to a variety of peptidergic cells. EMBO J. 5, 1495–1502 10.1002/j.1460-2075.1986.tb04388.x 3755681 PMC1166971

[85] Yoo, S.H. (1995) Purification and pH-dependent secretory vesicle membrane binding of chromogranin B. Biochemistry 34, 8680–8686 10.1021/bi00027a017 7612608

[86] Kang, Y.K. and Yoo, S.H. (1997) Identification of the secretory vesicle membrane binding region of chromogranin A. FEBS Lett. 404, 87–90 10.1016/s0014-5793(97)00099-9 9074643

[87] Yoo, S.H. and Kang, Y.K. (1997) Identification of the secretory vesicle membrane binding region of chromogranin B. FEBS Lett. 406, 259–262 10.1016/s0014-5793(97)00276-7 9136897

[88] Jentsch, T.J., Poet, M., Fuhrmann, J.C. and Zdebik, A.A. (2005) Physiological functions of CLC Cl- channels gleaned from human genetic disease and mouse models. Annu. Rev. Physiol. 67, 779–807 10.1146/annurev.physiol.67.032003.153245 15709978

[89] Comini, M., Sierra-Marquez, J., Guzman, G., Franzen, A., Willuweit, A., Katona, I. et al. (2022) CLC anion/proton exchangers regulate secretory vesicle filling and granule exocytosis in chromaffin cells. J. Neurosci. 42, 3080–3095 10.1523/JNEUROSCI.2439-21.2022 35241492 PMC8994546

[90] Guzman, R.E., Miranda-Laferte, E., Franzen, A. and Fahlke, C. (2015) Neuronal ClC-3 splice variants differ in subcellular localizations, but mediate identical transport functions. J. Biol. Chem. 290, 25851–25862 10.1074/jbc.M115.668186 26342074 PMC4646242

[91] Deriy, L.V., Gomez, E.A., Jacobson, D.A., Wang, X., Hopson, J.A., Liu, X.Y. et al. (2009) The granular chloride channel ClC-3 is permissive for insulin secretion. Cell Metab. 10, 316–323 10.1016/j.cmet.2009.08.012 19808024 PMC2778193

[92] Li, D.Q., Jing, X., Salehi, A., Collins, S.C., Hoppa, M.B., Rosengren, A.H. et al. (2009) Suppression of sulfonylurea- and glucose-induced insulin secretion in vitro and in vivo in mice lacking the chloride transport protein ClC-3. Cell Metab. 10, 309–315 10.1016/j.cmet.2009.08.011 19808023

[93] Maritzen, T., Keating, D.J., Neagoe, I., Zdebik, A.A. and Jentsch, T.J. (2008) Role of the vesicular chloride transporter ClC-3 in neuroendocrine tissue. J. Neurosci. 28, 10587–10598 10.1523/JNEUROSCI.3750-08.2008 18923035 PMC6671342

[94] Guzman, R.E., Grieschat, M., Fahlke, C. and Alekov, A.K. (2013) ClC-3 is an intracellular chloride/proton exchanger with large voltage-dependent nonlinear capacitance. ACS Chem. Neurosci. 4, 994–1003 10.1021/cn400032z 23509947 PMC3689194

[95] Park, E., Campbell, E.B. and MacKinnon, R. (2017) Structure of a CLC chloride ion channel by cryo-electron microscopy. Nature 541, 500–505 10.1038/nature20812 28002411 PMC5576512

[96] Fahlke, C. (2001) Ion permeation and selectivity in ClC-type chloride channels. Am. J. Physiol. Renal Physiol. 280, F748–F757 10.1152/ajprenal.2001.280.5.F748 11292616

[97] Jentsch, T.J., Maritzen, T., Keating, D.J., Zdebik, A.A. and Thevenod, F. (2010) ClC-3–a granular anion transporter involved in insulin secretion? Cell Metab. 12, 307–308 10.1016/j.cmet.2010.08.014 20889118

[98] Brown, E., Van Weering, J., Sharp, T., Mantell, J. and Verkade, P. (2012) Capturing endocytic segregation events with HPF-CLEM. Methods Cell Biol. 111, 175–201 10.1016/B978-0-12-416026-2.00010-8 22857929

[99] Hordejuk, R., Szewczyk, A. and Dolowy, K. (2006) The heterogeneity of ion channels in chromaffin granule membranes. Cell. Mol. Biol. Lett. 11, 312–325 10.2478/s11658-006-0027-1 16847559 PMC6472833

[100] Kelly, M.L., Abu-Hamdah, R., Jeremic, A., Cho, S.J., Ilie, A.E. and Jena, B.P. (2005) Patch clamped single pancreatic zymogen granules: direct measurements of ion channel activities at the granule membrane. Pancreatology 5, 443–449 10.1159/000086556 15985770

[101] Hu, Z.Z., Valencia, J.C., Huang, H., Chi, A., Shabanowitz, J., Hearing, V.J. et al. (2007) Comparative bioinformatics analyses and profiling of lysosome-related organelle proteomes. Int. J. Mass Spectrom. 259, 147–160 10.1016/j.ijms.2006.09.024 17375895 PMC1828028

[102] Faundez, V. and Hartzell, H.C. (2004) Intracellular chloride channels: determinants of function in the endosomal pathway. Sci. STKE 2004, re8 10.1126/stke.2332004re8 15150424

[103] Steinberg, B.E., Huynh, K.K., Brodovitch, A., Jabs, S., Stauber, T., Jentsch, T.J. et al. (2010) A cation counterflux supports lysosomal acidification. J. Cell Biol. 189, 1171–1186 10.1083/jcb.200911083 20566682 PMC2894458

[104] Graves, A.R., Curran, P.K., Smith, C.L. and Mindell, J.A. (2008) The Cl^−^/H^+^ antiporter ClC-7 is the primary chloride permeation pathway in lysosomes. Nature 453, 788–792 10.1038/nature06907 18449189

[105] Gasnier, C., Lugardon, K., Ruh, O., Strub, J.M., Aunis, D. and Metz-Boutigue, M.H. (2004) Characterization and location of post-translational modifications on chromogranin B from bovine adrenal medullary chromaffin granules. Proteomics 4, 1789–1801 10.1002/pmic.200300693 15174145

[106] Maji, S.K., Perrin, M.H., Sawaya, M.R., Jessberger, S., Vadodaria, K., Rissman, R.A. et al. (2009) Functional amyloids as natural storage of peptide hormones in pituitary secretory granules. Science 325, 328–332 10.1126/science.1173155 19541956 PMC2865899

[107] Westermark, P., Andersson, A. and Westermark, G.T. (2011) Islet amyloid polypeptide, islet amyloid, and diabetes mellitus. Physiol. Rev. 91, 795–826 10.1152/physrev.00042.2009 21742788

[108] Hosaka, M., Watanabe, T., Sakai, Y., Kato, T. and Takeuchi, T. (2005) Interaction between secretogranin III and carboxypeptidase E facilitates prohormone sorting within secretory granules. J. Cell Sci. 118, 4785–4795 10.1242/jcs.02608 16219686

[109] Prasad, P., Yanagihara, A.A., Small-Howard, A.L., Turner, H. and Stokes, A.J. (2008) Secretogranin III directs secretory vesicle biogenesis in mast cells in a manner dependent upon interaction with chromogranin A. J. Immunol. 181, 5024–5034 10.4049/jimmunol.181.7.5024 18802106

[110] Zhou, W., Yadav, G.P., Yang, X., Qin, F., Li, C. and Jiang, Q.X. (2022) Cryo-EM structure-based selection of computed ligand poses enables design of MTA-synergic PRMT5 inhibitors of better potency. Commun. Biol. 5, 1054 10.1038/s42003-022-03991-9 36192627 PMC9530242

[111] Iguchi, H., Bannai, S., Takanashi, N. and Tsukada, Y. (1992) Production of chromogranin A and B derived peptides in human small cell lung carcinoma cell lines. Eur. J. Cancer 28A, 1458–1462 10.1016/0959-8049(92)90543-b 1325178

[112] Vieau, D., Rojas-Miranda, A., Verley, J.M., Lenne, F. and Bertagna, X. (1991) The secretory granule peptides 7B2 and CCB are sensitive biochemical markers of neuro-endocrine bronchial tumours in man. Clin. Endocrinol. 35, 319–325 10.1111/j.1365-2265.1991.tb03543.x 1752059

[113] Nielsen, E., Welinder, B.S. and Madsen, O.D. (1991) Chromogranin-B, a putative precursor of eight novel rat glucagonoma peptides through processing at mono-, di-, or tribasic residues. Endocrinology 129, 3147–3156 10.1210/endo-129-6-3147 1954895

[114] Iguchi, H., Natori, S., Kato, K., Nawata, H. and Chretien, M. (1988) Different processing of chromogranin B into GAWK-immunoreactive fragments in the bovine adrenal medulla and pituitary gland. Life Sci. 43, 1945–1952 10.1016/s0024-3205(88)80013-4 3200116

[115] Benjannet, S., Leduc, R., Adrouche, N., Falgueyret, J.P., Marcinkiewicz, M., Seidah, N.G. et al. (1987) Chromogranin B (secretogranin I), a putative precursor of two novel pituitary peptides through processing at paired basic residues. FEBS Lett. 224, 142–148 10.1016/0014-5793(87)80438-6 3678488

[116] Benjannet, S., Leduc, R., Lazure, C., Seidah, N.G., Marcinkiewicz, M. and Chretien, M. (1985) GAWK, a novel human pituitary polypeptide: isolation, immunocytochemical localization and complete amino acid sequence. Biochem. Biophys. Res. Commun. 126, 602–609 10.1016/0006-291x(85)90648-5 3970711

[117] Delevoye, C., Marks, M.S. and Raposo, G. (2019) Lysosome-related organelles as functional adaptations of the endolysosomal system. Curr. Opin. Cell Biol. 59, 147–158 10.1016/j.ceb.2019.05.003 31234051 PMC6726539

[118] Marks, M.S., Heijnen, H.F. and Raposo, G. (2013) Lysosome-related organelles: unusual compartments become mainstream. Curr. Opin. Cell Biol. 25, 495–505 10.1016/j.ceb.2013.04.008 23726022 PMC3729921

[119] Binotti, B., Pavlos, N.J., Riedel, D., Wenzel, D., Vorbruggen, G., Schalk, A.M. et al. (2015) The GTPase Rab26 links synaptic vesicles to the autophagy pathway. eLife 4, e05597 10.7554/eLife.05597 25643395 PMC4337689

[120] Brunner, Y., Coute, Y., Iezzi, M., Foti, M., Fukuda, M., Hochstrasser, D.F. et al. (2007) Proteomics analysis of insulin secretory granules. Mol. Cell. Proteomics 6, 1007–1017 10.1074/mcp.M600443-MCP200 17317658

[121] Jacobson, D.A. and Philipson, L.H. (2007) Action potentials and insulin secretion: new insights into the role of Kv channels. Diabetes Obes. Metab. 9, 89–98 10.1111/j.1463-1326.2007.00784.x 17919183 PMC5878925

[122] Barnett, D.W., Pressel, D.M. and Misler, S. (1995) Voltage-dependent Na^+^ and Ca^2+^ currents in human pancreatic islet beta-cells: evidence for roles in the generation of action potentials and insulin secretion. Pflugers Arch. 431, 272–282 10.1007/BF00410201 9026789

[123] Fuchsberger, C., Flannick, J., Teslovich, T.M., Mahajan, A., Agarwala, V., Gaulton, K.J. et al. (2016) The genetic architecture of type 2 diabetes. Nature 536, 41–47 10.1038/nature18642 27398621 PMC5034897

[124] Barg, S., Eliasson, L., Renstrom, E. and Rorsman, P. (2002) A subset of 50 secretory granules in close contact with L-type Ca^2+^ channels accounts for first-phase insulin secretion in mouse beta-cells. Diabetes 51, S74–S82 10.2337/diabetes.51.2007.s74 11815462

[125] Best, L. (2005) Glucose-induced electrical activity in rat pancreatic beta-cells: dependence on intracellular chloride concentration. J. Physiol. 568, 137–144 10.1113/jphysiol.2005.093740 16024506 PMC1474780

[126] Singh, H., Cousin, M.A. and Ashley, R.H. (2007) Functional reconstitution of mammalian ‘chloride intracellular channels’ CLIC1, CLIC4 and CLIC5 reveals differential regulation by cytoskeletal actin. FEBS J. 274, 6306–6316 10.1111/j.1742-4658.2007.06145.x 18028448

[127] Ashley, R.H. (2003) Challenging accepted ion channel biology: p64 and the CLIC family of putative intracellular anion channel proteins (Review). Mol. Membr. Biol. 20, 1–11 10.1080/09687680210042746 12745921

[128] Yazawa, M., Ferrante, C., Feng, J., Mio, K., Ogura, T., Zhang, M. et al. (2007) TRIC channels are essential for Ca^2+^ handling in intracellular stores. Nature 448, 78–82 10.1038/nature05928 17611541

[129] Foskett, J.K., White, C., Cheung, K.H. and Mak, D.O. (2007) Inositol trisphosphate receptor Ca^2+^ release channels. Physiol. Rev. 87, 593–658 10.1152/physrev.00035.2006 17429043 PMC2901638

[130] Marius, P., Guerra, M.T., Nathanson, M.H., Ehrlich, B.E. and Leite, M.F. (2006) Calcium release from ryanodine receptors in the nucleoplasmic reticulum. Cell Calcium 39, 65–73 10.1016/j.ceca.2005.09.010 16289270

[131] Yoo, S.H., You, S.H., Kang, M.K., Huh, Y.H. and Shim, C.S. (2002) Localization of secretory granule marker protein chromogranin B in the nucleus. Ann. N. Y. Acad. Sci. 971, 345–348 10.1111/j.1749-6632.2002.tb04492.x 12438148

[132] Yoo, S.H., You, S.H., Kang, M.K., Huh, Y.H., Lee, C.S. and Shim, C.S. (2002) Localization of the secretory granule marker protein chromogranin B in the nucleus. Potential role in transcription control. J. Biol. Chem. 277, 16011–16021 10.1074/jbc.M105594200 11854265

[133] Yang, Z., Kirton, H.M., MacDougall, D.A., Boyle, J.P., Deuchars, J., Frater, B. et al. (2015) The Golgi apparatus is a functionally distinct Ca^2+^ store regulated by the PKA and Epac branches of the beta1-adrenergic signaling pathway. Sci. Signal. 8, ra101 10.1126/scisignal.aaa7677 26462734 PMC4869832

[134] Idevall-Hagren, O. and Tengholm, A. (2020) Metabolic regulation of calcium signaling in beta cells. Semin. Cell Dev. Biol. 103, 20–30 10.1016/j.semcdb.2020.01.008 32085965

[135] Vassileva, K., Marsh, M. and Patel, S. (2020) Two-pore channels as master regulators of membrane trafficking and endocytic well-being. Curr. Opin. Physiol. 17, 163–168 10.1016/j.cophys.2020.08.002 32838099 PMC7426208

[136] Yoo, S.H., Kang, M.K., Kwon, H.S., Lee, J.S., So, S.H., Ahn, T. et al. (2000) Inositol 1,4,5-trisphosphate receptor and chromogranins A and B in secretory granules. Co-localization and functional coupling. Adv. Exp. Med. Biol. 482, 83–94 10.1007/0-306-46837-9_6 11192603

[137] Accardi, A. and Miller, C. (2004) Secondary active transport mediated by a prokaryotic homologue of ClC Cl− channels. Nature 427, 803–807 10.1038/nature02314 14985752

[138] Sawicka, M. and Dutzler, R. (2022) Regulators of cell volume: the structural and functional properties of anion channels of the LRRC8 family. Curr. Opin. Struct. Biol. 74, 102382 10.1016/j.sbi.2022.102382 35504105

[139] Jentsch, T.J., Lutter, D., Planells-Cases, R., Ullrich, F. and Voss, F.K. (2016) VRAC: molecular identification as LRRC8 heteromers with differential functions. Pflugers Arch. 468, 385–393 10.1007/s00424-015-1766-5 26635246

[140] Voss, F.K., Ullrich, F., Munch, J., Lazarow, K., Lutter, D., Mah, N. et al. (2014) Identification of LRRC8 heteromers as an essential component of the volume-regulated anion channel VRAC. Science 344, 634–638 10.1126/science.1252826 24790029

[141] Willig, K.I., Rizzoli, S.O., Westphal, V., Jahn, R. and Hell, S.W. (2006) STED microscopy reveals that synaptotagmin remains clustered after synaptic vesicle exocytosis. Nature 440, 935–939 10.1038/nature04592 16612384

[142] Holroyd, P., Lang, T., Wenzel, D., De Camilli, P. and Jahn, R. (2002) Imaging direct, dynamin-dependent recapture of fusing secretory granules on plasma membrane lawns from PC12 cells. Proc. Natl Acad. Sci. U.S.A. 99, 16806–16811 10.1073/pnas.222677399 12486251 PMC139225

